# Immunization of Rabbits with Highly Purified, Soluble, Trimeric Human Immunodeficiency Virus Type 1 Envelope Glycoprotein Induces a Vigorous B Cell Response and Broadly Cross-Reactive Neutralization

**DOI:** 10.1371/journal.pone.0098060

**Published:** 2014-05-20

**Authors:** Gerald V. Quinnan, Olusegun Onabajo, Pengfei Zhang, Lianying Yan, Joseph J. Mattapallil, Zhiqiang Zhang, Ming Dong, Min Lu, David Montefiori, Celia LaBranche, Christopher C. Broder

**Affiliations:** 1 Department of Preventive Medicine and Biometrics, Uniformed Services University of the Health Sciences, Bethesda, Maryland, United States of America; 2 Department of Microbiology and Immunology, Uniformed Services University of the Health Sciences, Bethesda, Maryland, United States of America; 3 Department of Microbiology and Molecular Genetics, Public Health Research Institute, New Jersey Medical School, Newark, New Jersey, United States of America; 4 Department of Surgery, Duke University Medical Center, Durham, North Carolina, United States of America; CIP, NCI-Frederick, NIH, United States of America

## Abstract

Previously we described induction of cross-reactive HIV-1 neutralizing antibody responses in rabbits using a soluble HIV-1 gp140 envelope glycoprotein (Env) in an adjuvant containing monophosphoryl lipid A (MPL) and QS21 (AS02_A_). Here, we compared different forms of the same HIV-1 strain R2 Env for antigenic and biophysical characteristics, and in rabbits characterized the extent of B cell induction for specific antibody expression and secretion and neutralizing responses. The forms of this Env that were produced in and purified from stably transformed 293T cells included a primarily dimeric gp140, a trimeric gp140 appended to a GCN4 trimerization domain (gp140-GCN4), gp140-GCN4 with a 15 amino acid flexible linker between the gp120 and gp41 ectodomain (gp140-GCN4-L), also trimeric, and a gp140 with the flexible linker purified from cell culture supernatants as either dimer (gp140-L(D)) or monomer (gp140-L(M)). Multimeric states of the Env proteins were assessed by native gel electrophoresis and analytical ultracentrifugation. The different forms of gp140 bound broadly cross-reactive neutralizing (BCN) human monoclonal antibodies (mAbs) similarly in ELISA and immunoprecipitation assays. All Envs bound CD4i mAbs in the presence and absence of sCD4, as reported for the R2 Env. Weak neutralization of some strains of HIV-1 was seen after two additional doses in AS02_A_. Rabbits that were given a seventh dose of gp140-GCN4-L developed BCN responses that were weak to moderate, similar to our previous report. The specificity of these responses did not appear similar to that of any of the known BCN human mAbs. Induction of spleen B cell and plasma cells producing immunoglobulins that bound trimeric gp140-GCN4-L was vigorous, based on ELISpot and flow cytometry analyses. The results demonstrate that highly purified gp140-GCN4-L trimer in adjuvant elicits BCN responses in rabbits accompanied by vigorous B cell induction.

## Introduction

Induction of antibodies that neutralize many strains of human immunodeficiency virus type 1 (HIV-1) cross-reactively is a major goal of HIV-1 vaccine development efforts. The reasons for difficulty in achieving this goal are numerous, and include extreme genetic variability of the Env genes and the ability of the virus to shield critical epitopes through various structural mechanisms. Efforts to induce potent, broadly cross-reactive HIV-1 neutralizing antibodies (bNab) have included many approaches, none of which have been highly successful. The need for such responses is highlighted by results of clinical trials of HIV-1 Env-based vaccine candidates that induced weak nAb with little cross reactivity and that resulted in either no protection or short term protection of the minority of vaccinees in the trial[1], [2]. Furthermore, vaccine approaches that emphasize induction of cellular immunity have not generally resulted in complete protection from infection in non-human primate models, and in one clinical trial vaccinated individuals were more likely to become infected than controls[3]. Recent reports of recovery of broadly cross-neutralizing human monoclonal antibodies (mAbs) from infected individuals with bNab responses have greatly enhanced understanding of epitopes that induce such responses[4]–[8]. These observations have engendered optimism that approaches may be found to induce potent, protective bNab by vaccination[9].

In previous reports we have described induction of cross reactive nAb using immunization regimens that incorporate a particular HIV-1 Env, designated R2[10]–[12]. This Env was obtained from an HIV-1 infected patient with bNab a number of years ago[13]. The first immunogenicity studies conducted with R2 Env involved initial immunizations with Venezuelan equine encephalitis virus replicons that expressed the R2 Env in vivo, followed by a series of doses of soluble R2 gp140 in lipid-based adjuvant[10]. Using this approach moderately cross-reactive nAb were induced in small animals and non-human primates; those primates with moderately potent nAb against a recombinant Simian-Human Immunodeficiency virus were completely protected against intravenous challenge with that virus. In a subsequent study rabbits were immunized with the same R2 gp140 in the GlaxoSmithKline Biologicals (GSK) proprietary adjuvant, AS02_A_ [14]. In this study bNab were induced, although the potency of the responses was generally low. The soluble gp140 used in those studies comprised R2 gp120 fused in sequence to the gp41 ectodomain as a result of mutation of the furin protease site that normally at which gp160 is normally cleaved into its subunits. The gp140 was produced in non-human primate cell culture infected with recombinant vaccinia virus expressing the modified Env gene. Although the gp140 released by lysis of the infected cells was extensively purified, the immunogen was still contaminated with cellular proteins that induced antibodies reactive with human cell proteins present on viruses tested in neutralization assays. The gp140 produced using this method was predominantly dimeric, with some trimer and less monomer. The gp140 produced in this fashion generally binds mAbs and undergoes CD4-induced conformational changes similarly to native viral Env. However, since the majority of the gp140 molecules were not trimeric, there is concern that epitopes important for bNab may not be well presented by the non-native forms of the protein. The present study was designed and conducted to confirm the immunogenicity of the R2 gp140 in GSK adjuvant in rabbits, to address considerations regarding purity of the Env immunogen, and to develop further understanding of the responses induced in this rabbit model.

The gp140 used in the present study was produced using stably transformed cell lines, such that the level of contamination with cell proteins after purification was dramatically reduced compared to the vaccinia-derived protein used in previous studies. The multimeric state of the protein was characterized by biophysical methods, and antigenic reactivity characterized using various mAbs. The gp140 was produced as four different forms. One form (gp140) was of the same sequence as that used previously, and was purified so that the monomeric, dimeric, and trimeric forms were retained in the immunogen, as previously. A second form was produced as trimeric protein by virtue of in-frame fusion of the R2 gp140 coding sequence to a modified GCN4 multimerization domain (gp140-GCN4) [15], [16]. The third form was similar to the second, but had a flexible linker sequence introduced between the gp120 and gp41 coding sequences of R2 Env (gp140-GCN4-L) [17]. The rationale for the linker was that the flexibility that it allows might permit the trimeric proteins to assume more native state than would be possible for the uncleaved gp140. The fourth form consisted of gp140 with the linker sequence but no trimerization domain (gp140-L). All immunizations were administered in adjuvant provided by GSK. The results confirm the induction of an Ab response to Env that neutralizes otherwise resistant primary viruses cross-reactively.

## Materials and Methods

### Production of Env Glycoproteins

The gp140 used in the immunization experiments was produced using the phCMVhygro expression system that we have used previously. Codon optimized R2 *env* gene was cloned into the vector, which was modified from the plasmid phCMV (Genlantis, Inc) by introduction of a hygromycin resistance gene. phCMV uses a CMV promoter and enhancer driven cassette. In addition, upstream from the insert, in the promoter region, is an intron sequence and downstream is an efficient artificial terminator to ensure high transcription levels. This vector has been used for production of a stably transformed cell line that produces mg quantities of Nipah and Hendra virus glycoproteins[18], [19].

The gp140 coding sequence was prepared by insertion of two translational termination codons following the lysine residue at amino acid position 693, just prior to the predicted gp41 transmembrane region. The gp140 construct was also modified to contain arginine to serine substitutions at positions 517 and 520 to disrupt the protease cleavage signal to increase the yield of oligomeric Env during production[11]. The gp140 sequence was further modified in three ways. First, gp140-L was prepared by insertion during synthesis of the sequence encoding the flexible linker, GGGGSGGGGSGGGGS, in place of the protease cleavage site. This is the same linker sequence described by Chow et al[17]. Second, the gp140-GCN4 and gp140-GCN4-L sequences were prepared by ligating the modified GCN4 dimerization domain in frame to the 3′ terminus of the coding sequences of gp140 and gp140-L. This modification is expected to yield stable trimers. An Avitag sequence, encoding amino acids LNDIFEAQKIEWHE, was added to the 3′ end of the gp140-GCN4-L coding sequence for preparation of protein for use in flow cytometry studies.

To produce stably transformed cell lines, 293T cells were transfected with the respective constructs in 6-well cell culture plates, and the supernatant medium was tested for protein expression, and then replaced with medium containing hygromycin. The cells were passaged three times, then passaged at limiting dilution in 96 well plates for cloning. Cells producing high levels of protein were expanded and subjected to a second round of limiting dilution. Clones with the highest expression levels were selected for use in production. Production cell cultures were established in roller bottles, with yield per bottle of approximately 0.5–1.0 mg, or hollow-fiber cartridges, with yields up to 100 mg over several weeks of production.

Cell line culture supernatants (approximately 1 liter) were collected, centrifuged, and filtered through a 0.22 µM membrane. The cleared supernatant containing gp140 was applied to an XK26 column containing 25 ml bed volume of lentil lectin Sepharose 4B equilibrated with PBS, 0.5% Triton X-100, 0.02 *M* Tris-HCl, pH 7.5. The lentil lectin sepharose was then washed with 10x bed volume of PBS, 0.5% Triton X-100, 0.3 *M* NaCl, 0.02 *M* Tris-HCl, pH 7.5 followed by 4 bed volume of PBS, 0.02 *M* Tris-HCl, pH 7.5. Bound gp140 was then eluted with PBS, 0.02 *M* Tris-HCl, pH 7.5, 0.5 *M* methyl α-D-mannopyranoside. The eluted protein was concentrated and buffer exchanged with PBS using Centriprep concentrators to approximately 5–10 mls. The concentrated protein was diluted to 150 ml in PBS, and then loaded onto a Capto-DEAE (30 ml) column (GE Healthcare) equilibrated with 20 mM Tris, 100 mM NaCl, pH 8.0. Under these conditions, gp140 does not bind to the column, but contaminating proteins are retained on the column. The gp140 containing flow-through was concentrated again to approximately 2∼3 ml.

The concentrated gp140 was then subjected to size exclusion chromatography using a HiLoad 16/60 Superdex 200 prep grade gel filtration column (GE healthcare). Fractions of ∼1000 µl were collected. 10 µl and 1 µl of each fraction were analyzed using the Blue Native gel electrophoresis system (Invitrogen) for both Coomassie staining and Western blot detection. For immunodetection in Western blot, polyclonal rabbit anti-gp140 serum R2143 was used.

The apparent trimer and dimer fractions were then pooled and, typically, further concentration was not required. Observed concentrations were 0.6–2.2 mg/ml. The material was then sterile filtered, aliquoted, and frozen at −80°C. Aggregation following freezing at −80°C and subsequent thawing was not observed.

### Evaluation of Antigenic Reactivity of gp140

The gp140, gp140-GCN4, gp140-GCN4-linker, and gp140-linker were compared by immunoprecipitation in the presence and absence of four domain soluble CD4 (Progenics Pharmaceuticals, Inc., Tarrytown, NY). The mAbs used were gifts from James Robinson, Tulane University (mAbs A32, E51, 17b, 48d, 48e, 23e, 411g (411g is an IgG1, CD4i mAb, personal communication, James Robinson) and 49e), and Jonathan Gershoni, Univerity of Tel Aviv (CG10) [20]–[26].

Each of the different forms of gp140 was incubated with each of the mAbs for 4 hr at 4°C, followed by Protein G Sepharose (50 µl, 20% solution) precipitation for an additional 2 h at 4°C. The precipitated complexes were washed five times with lysis buffer, 5 min each time at 4°C, and then resuspended in SDS-PAGE sample buffer. The samples were boiled for 5 min and resolved on 4–12% Bis-Tris SDS-PAGE, followed by western blotting using R2143 polyclonal serum for detection. Gels were evaluated by densitometry.

### R2gp140 Co Receptor Binding Assay

Different versions of purified R2 gp140 were tested for their ability to associate with co-receptor CCR5 in the presence or absence of sCD4 receptor. In this assay, CCR5 expressing canine thymocyte Cf2ThCCR5 cells were used as the source of co- receptor[27]. The expressed CCR5 has a C9 epitope tag that is detectable by mAb 1D4 [28] . The parental Cf2Th cell line was used as the negative control. Cf2ThCCR5 or Cf2Th cells were grown to confluence in 150 cm^2^ flasks. The culture medium was removed and washed once with PBS. Then, the cells were disassociated from the flask surface after 5–10 min incubation in 5 ml of 1 mM EDTA. The cell suspension was centrifuged and the pellet was resuspended in 10 ml PBS. The total number of cells was then determined using a hematocytometer. The cell suspension was pelleted and lysed in buffer containing 1% cymal-5, 150 mM NaCl, 20 mM Tris-HCl, 2 mM EDTA, 10% glycerol, and complete protease inhibitor cocktail tablet (Roche). 1.5 µg amounts of the different versions of purified R2 gp140 were pre-incubated with or without 1.5 µg of sCD4 for at least 2 hr at 4°C. Then, 1×10^6^ Cf2ThCCR5 cells in 700 µl lysis buffer were added to the gp140-CD4 complex or gp140 without CD4 as control. Another reaction with same the volume and amount of Cf2Th cells only was used as control. The reactions were incubated at 4°C for overnight. The next day, 1 µg of 1D4 was added to each reaction and the reaction was incubated for another 4 hrs followed by Protein G Sepharose (50 µl, 20% solution) precipitation for an additional 2 h at 4°C. The precipitated complexes were washed five times with lysis buffer, 5 mins each time at 4°C, and then resuspended in SDS-PAGE sample buffer and processed for Western blotting, as above. The blots were then probed for the presence of gp140, CCR5, and CD4 separately using polyclonal rabbit anti gp140 antiserum (R2143), polyclonal goat anti-CCR5 CKR5 (C-20) antibody, and a polyclonal anti-huCD4 affinity purified Goat IgG.

### Rabbit Immunizations

Animal studies were performed at Spring Valley Laboratories, Sykesville, MD, in accord with applicable ALAAC requirements for animal care and with approval of the Spring Valley Laboratories Institutional Animal Care and Use Committee, SVL-045. Immunizations were carried out in NZW rabbits. Each construct was studied in a group of five rabbits. All groups of rabbits were given 30 mcg gp140 in 0.5 ml of adjuvant on weeks 0, 4, 8, and 26, and on months 8 and 10. The controls and the group receiving gp140-GCN4-L were given a final dose at month 12. In the previous study by Zhang et al, R2 gp140 was administered in AS02_A_ adjuvant [14]. Based on results obtained in other studies since that study was initiated, a decision was made to use the adjuvant AS01_B_ in this study. AS02_A_ and AS01_B_ contain the same key active ingredients formulated differently. AS02_A_ is an Adjuvant System containing MPL, QS-21 in an o/w emulsion (50 µg MPL and 50 µg QS-21). AS01_B_ is an Adjuvant System containing MPL, QS-21 and liposome (50 µg MPL and 50 µg QS-21). After observing the neutralization induced by the first four doses in this study, the adjuvant for the last three doses was changed to AS02_A_. Sera were collected before immunization and 10 days after each immunization. Rabbits were euthanized by exsanguination under general anesthesia by injection of 60 mg/Kg ketamine and 5 mg/Kg xylazine via I.M. In some cases spleens were removed and single cell suspensions prepared at necropsy. Cell suspensions were stored in liquid nitrogen for later use.

#### Viruses and neutralization assays

The pseudotyped virus neutralization assay procedure described by Zhang et al was used[13]. Briefly, envelope glycoprotein genes corresponding to the various virus strains, were used to prepare pseudotypes by cotransfection of 293T cells with pNL4-3E-R-.luc[14]. The R2 strain is neutralization sensitive, and we consider it to be Tier 1. The strains SF162, SF162-K160N, and SF162-N332A were kindly provided by L. Stamatatos[29]. The other strains were considered to be Tier 1b, 2 or 3, according to Li et al[30]. Neutralization assays were performed in HOS.CD4−CCR5 cells or TZM-BL cells, testing sera at serial two-fold dilutions beginning at 1∶5, using methods previously described. In the HOS.CD4−CCR5 cell assay a neutralization titer was considered to be the highest dilution that inhibited luciferase activity by more than 50% in comparison to sera from concurrent bleeds of control rabbits tested in the same assays. In the TZM-BL cell based assay neutralization endpoints were calculated by regression analysis [14], [30]–[32].

Blocking assays to determine the presence of bNab targeting the CD4 binding site (CD4bs) were performed using RSC3 and RSC3-371I as control[33]. To determine the presence of antibodies targeting the V1/V2 region, gp70(MLV)-V1V2(HIV-1/CN54) (gp70-V1V2) was used (Immune Technology Corp, New York, NY). The gp70-V1V2 was used for measuring serum antibodies in ELISA[34].

### Serum Absorptions

Cells obtained by trypsinization from a confluent 75 cm^2^ flask of 293T cells were resuspended in 400 µl of sera at final serum dilutions of 1∶2.5. The suspensions were incubated at 4°C for 3 h with light rocking, cells were sedimented by centrifugation, and the absorption was repeated with new cells a second and third time. The third absorption was continued overnight.

### Biotinylation of R2gp140

Trimeric R2gp140-GCN4-A with the Avitag Biotinylation signal LNDIFEAQKIEWHE was biotinylated using Biotin ligase (Avidity, Denver, Co) according to manufacturer’s directions. Details are as follows; 100 µl of Trimeric R2gp140 at a concentration of 1.6 mg/ml was mixed with 12.5 µl Biomix A 10X solution and 12.5 µl Biomix B 10X solution. 2.5 µl of BirA (biotin ligase 1 mg/ml) was added and the mixture was incubated at 30°C for 40 minutes. Biotinylated product was titrated using flow cytometry and stored at −20°C.

### Identification of gp140-specific B Cells

Rabbit PBMC were purified from peripheral blood by Ficoll-Paque density gradient centrifugation according to the manufacturer’s instructions. Purified PBMC were labeled with biotinylated trimeric R2gp140 at room temperature at a concentration of 0.5 µg/100 µl. Cells were washed 2X with PBS and labeled with anti-Biotin-APC (Miltenyi Biotec, Cambridge, MA), anti-Rabbit CD4-FITC; Clone Ken-4(Rockford IL) and Anti-Rabbit IgG-PE (Acris antibodies, San Diego, CA). All antibodies were titrated using Rabbit PBMC. Cells were analyzed using Becton Dickinson LSR II flow cytometer. Flow cytometric data was analyzed using FlowJo version 9.2 (Tree Star, Inc., Ashland, OR).

### ELISPOT Assays

Milipore EL ISpot plates (MAIPSWU) were used according to manufacturer’s instructions. For detection of total IgG producing cells, 100,000 cells per well were used, and detection was accomplished using Biotinylated Mouse Anti-Rabbit IgG (BD Pharmingen, San Diego, CA). For detection of anti-gp140 antibody-producing B cells, 200,000 cells were seeded per well and detection was accomplished using biotinylated R2gp140, as described above. In both cases Streptavidin HRP was used for color development from TMB substrate. Cells were tested after 72 hours of stimulation with IL-2 (10 ng/ml) + R848 (1 µg/ml) and without prestimulation, per instructions. There was no significant effect of prestimulation (results not shown), so both sets of results were averaged together for presentation here.

### Analytical Ultracentrifugation

Sedimentation equilibrium measurements were carried out on an XL-A analytical ultracentrifuge (Beckman Coulter, Fullerton, CA) equipped with an An-60 Ti rotor at 20°C. Peptide samples were dialyzed overnight against ABS (pH 5.2), loaded at initial concentrations of 30, 100, and 300 µM, and analyzed at rotor speeds of 18,000 and 21,000 rpm. Data sets were fitted to a single-species model. Random residuals were observed in all cases.

## Results

### Oligomerization of Envs

**Figure 1:**
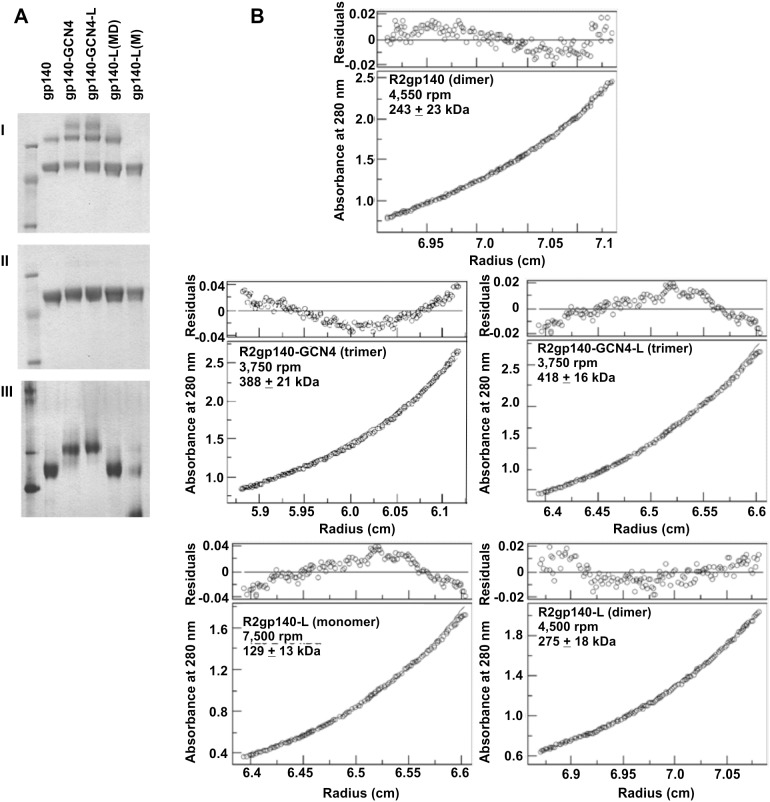
Purified oligomeric R2 HIV-1 envelope glycoproteins. (A) Three µg of each purified protein was loaded in each lane. Upper Panel: samples were treated with reducing SDS-PAGE sample buffer, boiled, separated by SDS-PAGE, and stained with coomassie blue. Lower Panel: samples were treated and separated using 3–12% Blue Native PAGE (In Vitrogen). Both the R2gp140-GCN4 and R2gp140- GCN4-L migrated as trimer with MW ∼720 kDa, whereas the wild-type R2gp140 migrated as >90% dimer with a MW of ∼520 kDa. (B) Size estimation of the differing forms of R2 gp140 was further estimated by analytical ultracentrifugation. The R2 gp140 preparations used in Figure 1A were subjected to sedimentation equilibrium analyses by ultracentrifugation. The identity of the gp140 form tested, centrifuge speed for equilibrium determination, and estimated molecular mass of each is shown in the inset in each panel.

The oligomeric states of the different Envs was characterized by PAGE and analytical ultracentrifugation. The results of PAGE are shown in Figure 1, Panel A. The upper gel in Panel A shows results of PAGE of denatured proteins under reducing conditions. The columns representing g140-L were fractions pooled from molecular sieve chromatography that were presumed, based on in process analyses, to include either both monomeric and dimeric (MD) forms or only dimeric (D). Under denaturing conditions all forms migrated as expected for gp140. In the lower part of Panel A is the gel obtained by electrophoresis under non-denaturing conditions. In this gel the predominant form of gp140 migrated as dimer, while gp140-GCN4 and gp140-GCN4-L each migrated as trimer with approximate molecular radii of 720 kDa. The gp140-L(D) migrated in parallel with gp140, and gp140-L(MD) did appear to contain both monomeric and dimeric forms. The size estimates for each of the species were confirmed by analytical ultracentrifugation, as shown in Figure 1B. The calculated sizes of each were fully consistent with the oligomeric state predicted by results of electrophoresis.

### Antigenic Reactivity of Oligomeric R2 gp140

**Figure 2:**
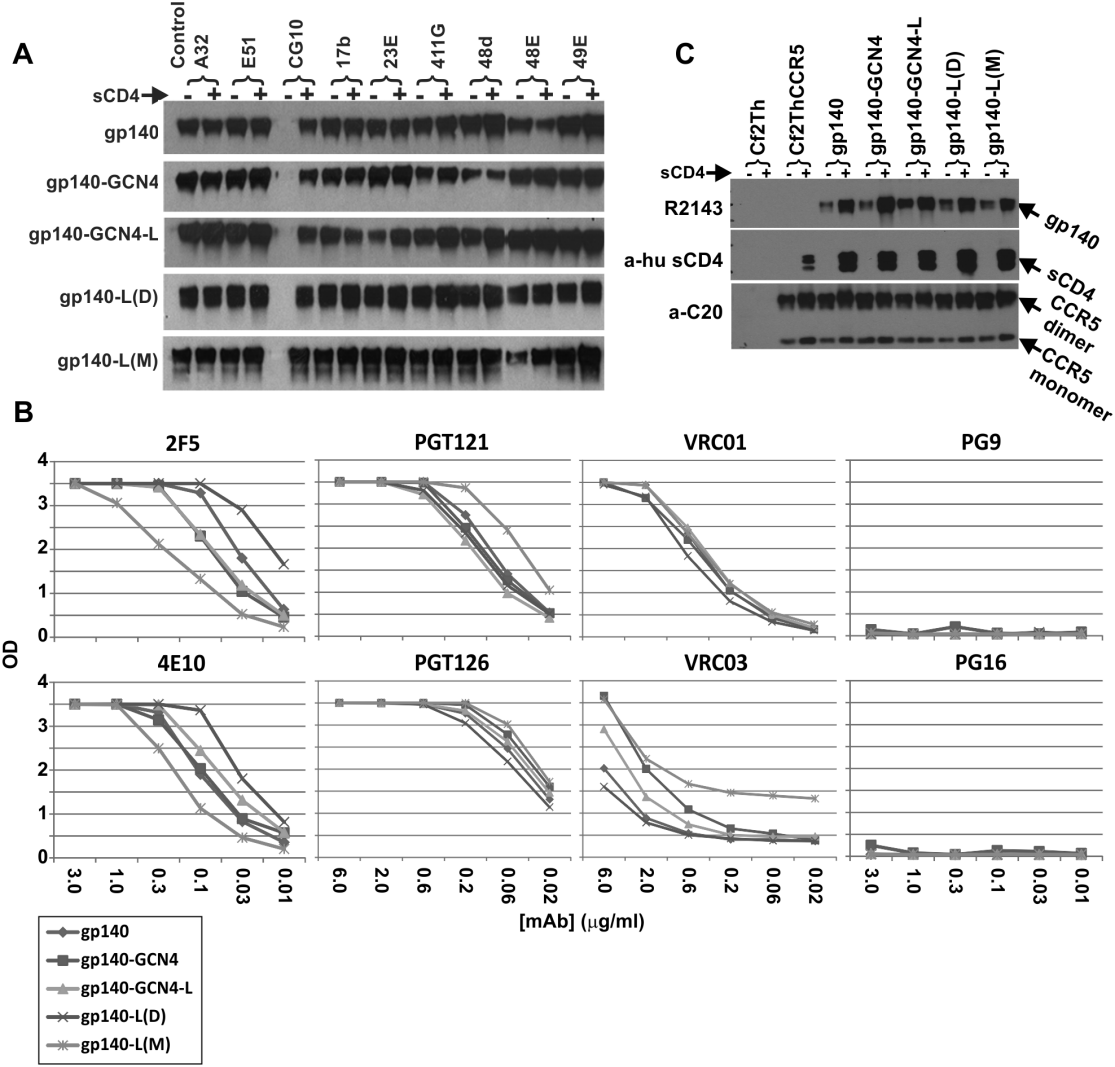
Antigenic reactivity and function of different forms of gp140. (A) Reactivity to CD4i and CD4-gp140 complex specific mAbs (left panel) and monoclonal antibody binding analysis (right panel) of purified R2 gp140(dimer), R2 gp140-GCN4 (trimer), R2 gp140-GCN4-L (trimer), R2gp140-L (dimer), and R2gp140-L (monomer). For the left panel, 1 µg of each of the different versions of purified R2 gp140 was incubated with or without excess (3 µg) sCD4 in 700 µl of reaction buffer (PBS containing 0.5% Triton X-100) at 4°C for overnight, followed by 2 µg of the indicated mAb for an additional 4 h. All mAbs bound all forms of the gp140 with or without sCD4, except mAb CG10, which probably binds a complex epitope formed by gp120 and CD4 residues. No significant enhancing effect of binding by sCD4 was noted. For the right panel, 1 µg of different versions of purified R2 gp140 was incubated with the indicated mAbs in 700 µl of reaction buffer for 4 h at 4°C. Each complex was then precipitated with 50 µl of Protein G Sepharose (20% solution) for an additional 2 h at 4°C. The precipitated complexes were washed, resuspended in SDS-PAGE sample buffer, boiled for 5 min and resolved on 4–12% Bis-Tris SDS-PAGE, and subjected to western blotting. The blots were probed with a polyclonal rabbit anti-gp140 antiserum. The mAbs included human mAbs that are known to neutralize HIV-1 cross-reactively, and murine mAbs (names beginning with “D”) used to probe for changes in epitope accessibility in the gp41 ectodomain. (B) Antigenic reactivity of different forms of gp140 with additional humAbs with broad neutralizing cross-reactivity was tested by immunoprecipitation and western blot. (C) Binding of co-receptor CCR5 to different versions of purified R2 gp140 was tested in the presence or absence of sCD4. The proteins tested are shown at the top. The canine thymocyte cell line, Cf2Th, served as a negative control, while the CCR5 expressing Cf2ThCCR5 cells were used as the source of coreceptor. After pre-incubation of the purified R2 gp140s with or without sCD4, the lysed Cf2ThCCR5 cells were applied for binding. After Protein G Sepharose precipitation, the proteins were resolved on 4–12% Bis-Tris SDS-PAGE followed by western blotting. The blots were probed for the presence of CCR5, gp140, and CD4 separately using polyclonal rabbit anti-gp140 antiserum, R2143 (upper panel), polyclonal anti-huCD4 affinity purified Goat IgG (middle panel), and polyclonal goat anti-CCR5 CKR5 (C-20) antibody (lower panel).

Shown in Figure 2A is specific mAb binding in the presence and absence of sCD4 assessed by immunoprecipitation (IP) followed by Western blot detection. We have shown previously that R2gp140 oligomer exhibits the ability to be recognized by CD4i mAbs both with and without CD4 binding, whereas other gp140 strains require CD4 binding for CD4i mAb binding to occur[14]. Figure 2A demonstrates the characteristic binding reactivity to a panel of CD4i human mAbs and of a CD4-gp120 epitope complex specific mAb, CG10. As we have previously observed, the binding of CD4 was not required for efficient interaction of any of the CD4i antibodies with R2g140 (wild-type). Similar results were obtained for gp140-GCN4, gp140-GCN-L, gp140-L-Monomer, and gp140-L-Dimer, indicating that CD4i epitopes are exposed on R2gp140 in all of the forms tested here. As expected, CG10 mAb reactivity was completely dependent on CD4 binding to the proteins, since CG10 recognizes the gp120-CD4 complex [25].

The results of mAb binding to the different proteins in ELISA are shown in Figure 2B. The mAbs 2F5 and 4E10 bind to a membrane proximal epitope region in gp41, both neutralize R2 (see below) and both bound the different forms of R2 well. The PGT121 and PGT126 mAbs bind to an N-linked glycan in V3 and other variable loop amino acids. They bound similarly to each of the forms of gp140. Both neutralize R2 strain well (see below). The VRC01 and VRC03 mAbs both target the CD4 binding site (CD4bs). VRC01 neutralizes the R2 strain well, while VRC03 neutralizes, but less well (see below). VRC01 bound all forms of gp140 similarly, while VRC03 varied in extent of binding, consistent with potentially lower affinity for the proteins. The PG9 and PG16 mAbs bind to a conformational epitope that involves positively charged amino acids in the beta sheet C region of gp120 and an N-linked glycan in the V2 loop. Neither of these mAbs bound well to any forms of the gp140 in immunoprecipitations. The lack of binding of these mAbs is consistent with the failure of these mAbs to neutralize the R2 strain (data not shown) and the sequence of R2, which lacks important positively charged residues and the glycosylation site in V2 important for binding.

### Gp140 Binding to CCR5

Proteins were compared for binding to CCR5 by immunoprecipitation in the presence and absence of sCD4, as shown in Figure 2C. The mixtures were immunoprecipitated using a mAb against an epitope tag on CCR5, and the blots were developed with antibodies to gp140, CD4, and CCR5. The Cf2Th cell line served as a negative control. No proteins were detected as precipitated from the control Cf2Th cell line (lanes 1 and 2). The lanes labeled Cf2ThCCR5 shows precipitation in the absence of gp140. The presence of sCD4 resulted in some increase in precipitation of CCR5, and a small amount of sCD4 was pulled down by the CCR5. Each of the forms of gp140 was pulled down in the absence of sCD4, and the amount was substantially increased in each case in the presence of sCD4. The amount of sCD4 that was pulled down was also increased in each case by the addition of gp140. These data generally confirmed the results in Figure 2A, indicating that R2gp140 binds CCR5 in the absence of sCD4; the enhanced binding of CCR5 in the presence of sCD4 is consistent with substantially greater infectivity of the R2 strain in CD4+ than CD4−negative cells. The results also demonstrated that the capacity of R2gp140 to form complexes with sCD4 and CCR5 was not diminished by the modifications we made.

### Neutralizing Antibody Responses to Immunization

**Figure 3:**
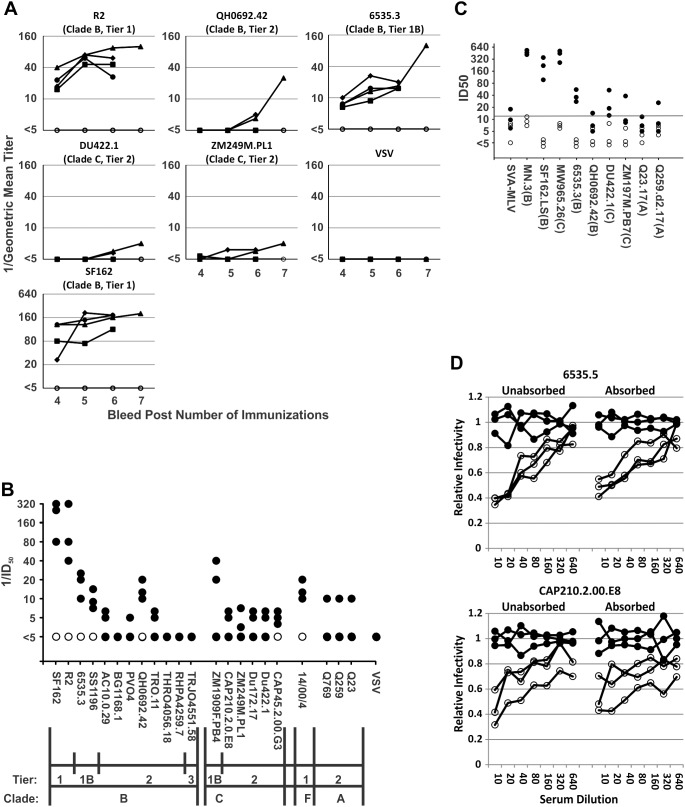
Neutralizing antibody responses after serial immunizations with different forms of R2gp140. Rabbits received immunizations containing 30 µg of wild type R2gp140 (⧫), R2gp140-GCN4 (▪), R2gp140-GCN4-L (▴), or R2gp140-L (•), or saline (○) mixed with adjuvant in 0.5 ml volumes divided subcutaneously in the hind legs. The first three immunizations were administered at 4 week intervals in the adjuvant AS01_B_. Immunizations four through seven were administered at weeks 26, 46, 53, and 69. The adjuvant was changed to AS02_A_ for the last three immunizations. Only three rabbits in the gp140-GCN4-L group and three controls received the seventh immunization. Rabbits were bled approximately 10 days after each immunization. (A) Results shown are geometric means for each immunization group, two separate assays for each rabbit. Sera were tested at serial dilutions beginning at 1∶5. Luminescence values obtained with test sera were compared to negative control sera to determine the highest dilution resulting in ≥50% neutralization, which was assigned as the endpoint. VSV-G protein pseudotyped virus was used as a specificity control. (B) Neutralizing responses of the three R2gp140-GCN4-L immunized (•) and three control (○) rabbits immunized were tested against an expanded panel of pseudotyped HIV-1 strains. Sera were tested at serial dilutions beginning at 1∶5. Results shown are geometric means of two or more separate assays. Luminescence results obtained with test sera were compared to results obtained in the presence of negative control sera. No neutralization was seen with the sera from sham immunized rabbits. The highest dilution of test serum that resulted in ≥50% inhibition of luminescence compared to control was designated as the 50% Inhibitory Dose (ID_50_). If a serum from an immunized rabbit showed no neutralization, the result is shown as <5. All results ≥5 are shown and points were nudged as necessary to allow for visualization of all results. The labels on the horizontal axis indicate the virus strain, Tier, and Clade for each virus tested. (C) Neutralization of HIV-1 strains in TZM-BL cell assay. Results are displayed similarly to panel B. Data points have been nudged for clarity. The horizontal line above the axis represents two standard deviations above the mean of all the results obtained using control sera. Clades of the viruses tested are shown parenthetically after each name below the horizontal axis. (D) Neutralizing activity of rabbit sera against a Clade B and a Clade C strain of HIV-1 was not affected by exhaustive absorption of sera with human cells. Assays were performed using the sera from after the seventh immunization. Cells obtained by trypsinization from a confluent 75 cm^2^ flask of 293T cells were resuspended in 400 µl of sera at final serum dilutions of 1∶2.5, incubated at 4°C for 3 h with light rocking, sedimented by centrifugation, and the absorption was repeated with new cells a second and third time. The third absorption was continued overnight. Results obtained with the three immune (○) and three control (•) sera against each of the two pseudotyped virus strains are shown as average results of two independent experiments.

The results of HOS-CD4+CCR5+ cell-based neutralization assays done after serial immunizations are presented in Figure 3A. After the first three immunizations little, if any neutralization of any strains tested was observed (data not shown). Immunizations in AS02_A_ adjuvant did result in induction of neutralizing responses. After dose 6 (2 doses using AS02_A_), responses measured were still low or absent against Tier 2 strains and low to modest against Tier 1 strains. There were greater numbers of rabbits with detectable neutralization in the gp140-GCN4-L group, although these differences were not statistically significant with respect to frequency or magnitude of responses. One additional immunization was given to three rabbits in the gp140-GCN4-L group, with further enhancement of neutralization after dose 7 against Tier 1b and Tier 2 strains. No neutralization of the control virus, VSV, was observed in any rabbit. The responses of each rabbit in this group against an expanded panel of pseudotyped strains in the HOS-CD4+CCR5+ cell assay are shown in Figure 3B. Sera were tested at serial dilutions beginning at 1∶5. Some inhibition of pseudotyped virus-induced luminescence, compared to treatment with medium alone, is commonly observed at dilutions of 1∶5 or 1∶10. Accordingly, luminescence results obtained with sera from immunized rabbits was compared to mean luminescence results obtained with control rabbit sera to determine whether inhibition occurred. As shown, inhibition greater than control was commonly observed with sera from the immunized, but not control rabbits. The neutralization sensitive strains SF162 and R2 (Clade B) and 14/00/4 (Clade F) were neutralized by all three immunized rabbits. Neutralization generally corresponded to sensitivity of the strains tested. Tier 1 and 1b strains were commonly neutralized at serum dilutions of 1∶10 or higher. Among Tier 2 strains, there was neutralization by one or more rabbits against 4/8 Clade B strains, 5/5 Clade C strains, and 3/3 Clade A strains. After completion of these assays, we recognized that the weak, but cross-reactive neutralization we observed in this study was similar to that observed in a previous study using R2gp140 produced using recombinant vaccinia virus infected cells; that is, the results obtained here after 2 and 3 doses of gp140 in AS02_A_ (total doses 6 and 7 in this study) were similar to the responses observed in the previous study after 3 and 4 doses. Similar responses were detected in the TZM-BL cell assay, as shown in Figure 3C. There was no neutralization of the control virus, SVA-MLV. There was moderate to strong neutralization of the three Tier 1 viruses, MN.3, SF162.LS, and MW965.26. There was weak to moderate neutralization of five of six Tier 1B or Tier 2, Clade A, B, or C strains.

The antigens used in this study were produced in the stably transformed human kidney cell line, 293T. We observed that the gp140 antigens produced by these stably transformed cells could be purified much more extensively than when produced by infection of cells with recombinant vaccinia virus, as we had done previously. To further assess whether the apparent neutralization observed in this study may have reflected the induction of anti-human, rather than anti-HIV-1 antibodies, we performed neutralization assays after exhaustive absorption of sera with the same cell line used to produce the pseudotyped viruses. These results are shown in Figure 3C. No significant reduction of neutralizing activity was noted after absorption against either the subtype B or C viruses tested. These results and the absence of neutralization of VSV, which was produced in the same cell line used to produce the HIV-1 pseudotypes, argue in support of the HIV-1 specificity of the neutralizing responses observed.

### Probing of Epitope Specificity of the Neutralizing Responses

**Figure 4:**
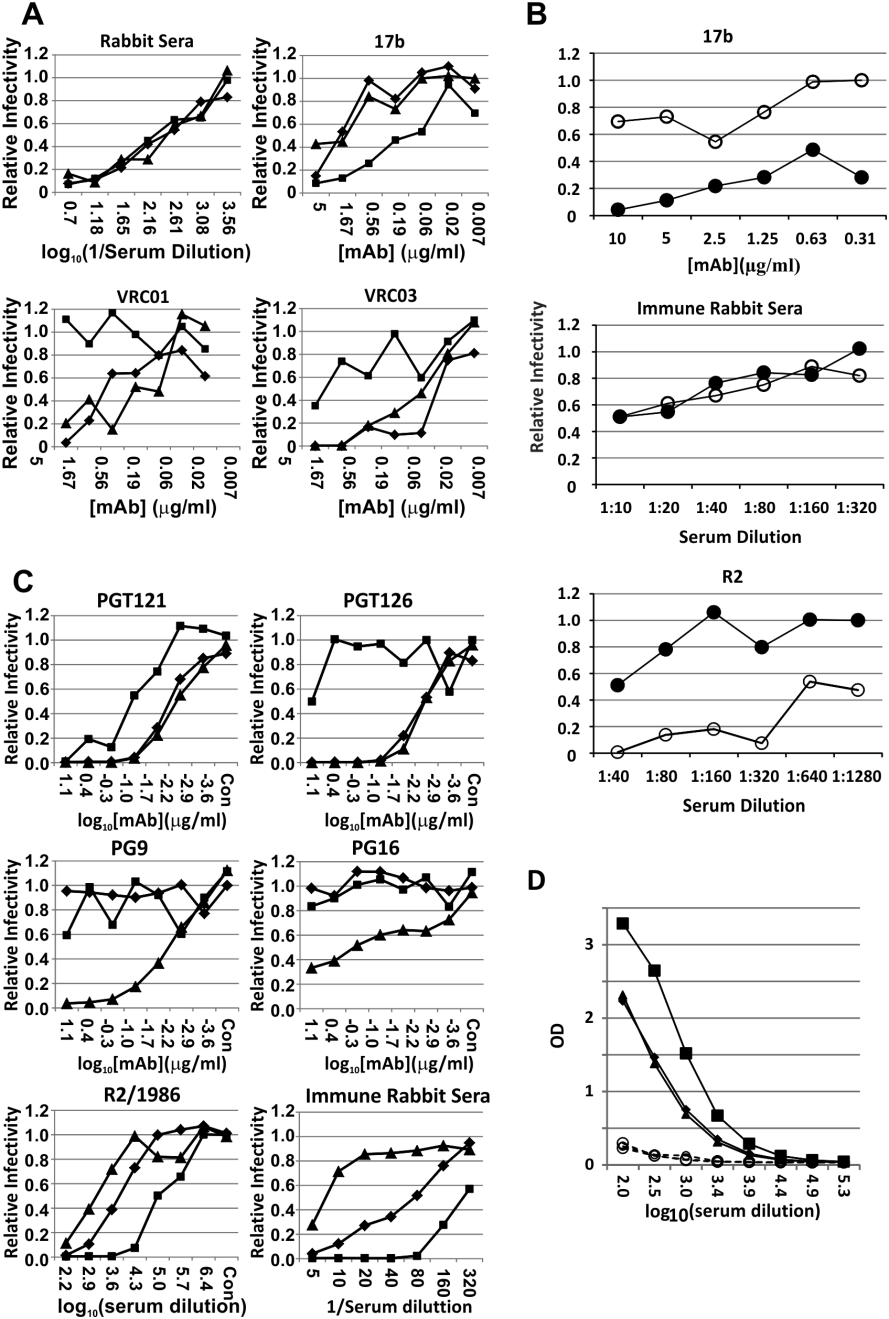
Probing the epitope specificity of neutralizing activity in sera from immunized rabbits. (A) Absence of blocking of neutralization by rabbit sera by the CD4 binding site scaffold RSC3. Neutralization of the R2 strain by post dose seven rabbit sera and mAbs 17b, VRC01, and VRC03 in the presence of medium alone (⧫), RSC3 (▪), or the CD4bs mutant scaffold RSC3Δ371I (▴) were compared. Each of the scaffold proteins were incubated with the sera for 1 hr at 37°C at 50 µg/ml before testing for neutralization. Results are representative of two separate experiments. Neutralization by mAb 17b was enhanced by the presence of RSC3, while neutralization by the CD4bs mAbs VRC01 and VRC03 was effectively abrogated by preincubation with RSC3. (B) Minimal effect of virus pre-binding by sCD4 on neutralization of HIV-2 by immune rabbit sera. Sera from after dose 7 from immune rabbits (#16, #17, #18) were tested for neutralization of pseudotyped HIV-2 in the presence (⧫) or absence (▪) of 1 µg/ml of sCD4. The CD4i mAb 17b and the R2 Serum were tested in comparison. Results are representative of three separate experiments. (C) Effect of V2 and V3 region glycans on neutralization by rabbit sera. Neutralization assays were performed against the wild type SF162 strain (⧫) and the N-linked glycosylation site mutant K160N (▴) and N332A (▪). Neutralization by PGT121 and PGT126 is known to be dependent upon the glycan at residue 332, with the expected result that there should be greater neutralization of wild type and K160N mutants than the N332A mutant. Neutralization by PG9 and PG16 is known to be dependent upon the glycan at residue 160, with the expected result that there should be greater neutralization of the K160N mutant than the other two. Note that serial dilutions for the mAbs and the serum from 1986 from the R2 donor were 1∶5, while the rabbit sera were serially diluted 1∶2. (D) ELISA assay of serum binding to the gp70V1/V2 scaffold fusion protein. The immunized rabbits are shown as solid lines with filled symbols, while the control rabbits are shown as open circles and dashed lines. Individual rabbits are: rabbit 16, ⧫; rabbit 17, ▪; rabbit 18, ▴.

Studies were performed to assess whether the antibodies induced were recognizing well-known cross-reactive neutralization epitopes. Broadly neutralizing human mAbs are often directed against the receptor, CD4, binding site. MAbs that neutralize through binding of epitopes in the CD4bs tend to bind with high affinity to the engineered gp120 derivative protein, RSC3[7]. Such high affinity is also observed for mAbs that only neutralize neutralization sensitive, Tier 1 strains. Blocking of neutralization by anti-CD4bs mAbs is also observed with RSC3. Conversely, binding of such mAbs is abrogated by the 371I mutation in RSC3Δ371I mutant. Experiments to test blocking of neutralization by rabbit sera by the RSC3 and RSC3Δ371I proteins are show in Figure 4A. The rabbit sera were compared to the mAbs 17b, VRC01, and VRC03. MAb 17b binds a CD4i epitope and mAbs VRC01 and VRC03 bind the CD4bs. Neutralization by 17b was not blocked by RSC3Δ371I, but was enhanced by RSC3, suggesting that the latter caused induction of the coreceptor binding site. Neutralization by VRC01 and VRC03 was inhibited by RSC3, but not by RSC3Δ371I, as expected. No effect on neutralization by either protein was observed for the rabbit sera. Based on these results it is unlikely that the cross-reactive neutralizing activity in the rabbit sera is directed against the CD4bs.

We next examined whether immunization of rabbits induced antibodies against coreceptor binding site epitopes on gp140, as shown in Figure 4B. Rabbit sera were tested for neutralization of HIV-2 Env pseudotyped virus in the presence and absence of sCD4. As described by Decker et al, HIV-2 Env is typically resistant to neutralization by antibodies induced by HIV-1, but can be made susceptible by first binding HIV-2 with sCD4, causing induction of coreceptor binding site epitopes (CD4i epitopes) [35]. Control testing included serum from the R2 donor and the mAb 17b. Sera from HIV-1 infected donors usually have CD4i antibodies and neutralize HIV-2 in the presence of sCD4. Similarly, mAb 17b is directed against the coreceptor binding site and neutralizes HIV-2 in the presence of sCD4 in this assay format. Neutralization by both R2 serum and mAb 17b were substantially enhanced in the presence of sCD4, while there was little if any suggestion of enhanced activity of the rabbit sera in the presence of sCD4. These results indicate that it is unlikely that the R2 gp140-GCN4-L induced significant neutralization targeting CD4i epitopes, even though the Env is distinctive in regards to its CD4 independence with respect to binding of CD4i epitopes and CCR5 (see above) and its ability to mediate CD4-independent infection.

Results of experiments testing for the presence of antibodies against glycan-dependent epitopes are shown in Figure 4C, which demonstrates neutralization of strain SF162 and the mutants SF162K160N and SF162N332A. The N332A mutation eliminates an N-linked glycosylation site important for neutralization by the mAbs PGT121 and PGT126. Thus, both SF162 and SF162K160N were well neutralized by these mAbs, but the N332A mutant was not neutralized or was neutralized less well than the others. The K160N mutation introduces an N-linked glycosylation site that is important for recognition by the mAbs PG9 and PG16. Accordingly, both SF162 and SF162N332A resisted neutralization by these two mAbs, while SF162K160N was neutralized well by them. In the lower panels of Figure 4C results are shown for serum obtained from the R2 donor and for the mean neutralizing results obtained for the post 7 dose rabbit sera. In both of these cases the results obtained were contrary to patterns observed in testing the mAbs. The SF162 mutant with both of the glycosylation sites present, K160N, was neutralized least, the wild type with only one of the sites present was neutralized at an intermediate level, and the N332A mutant lacking both sites was neutralized much more readily than the other two. Please note that the serial serum dilutions shown are five-fold. Thus, the 50% neutralization endpoints calculated by linear regression analyses were 5.0 and 121.7-fold greater for the SF162 wild type and SF162-N332A variant, respectively, than for the SF162-K160N variant. These results demonstrate that the neutralization of strain SF162 was not mediated by antibodies with specificities like PG9/16 or PGT121/126. If antibodies dependent upon these glycans are involved in neutralization of other strains, they do not cross-react with SF162.

Recent evidence indicates a correlation between vaccine efficacy in the Thai HIV vaccine efficacy trial and levels of antibody binding to various V1/V2 peptides[36]. The prototype assay for this activity involved binding to the scaffold fusion protein, gp70-V1/V2, developed by Pinter et al[37]. We evaluated the induction of antibodies targeting the V1/V2 region in ELISA assay using that scaffold fusion protein, as shown in Figure 4D. Each of the three immunized rabbits developed substantial antibody responses binding the V1/V2 fusion peptide.

### Quantification of Antibody Secreting Cells and B Cells Binding gp140 in Spleens and Mesenteric Lymph Nodes

**Figure 5:**
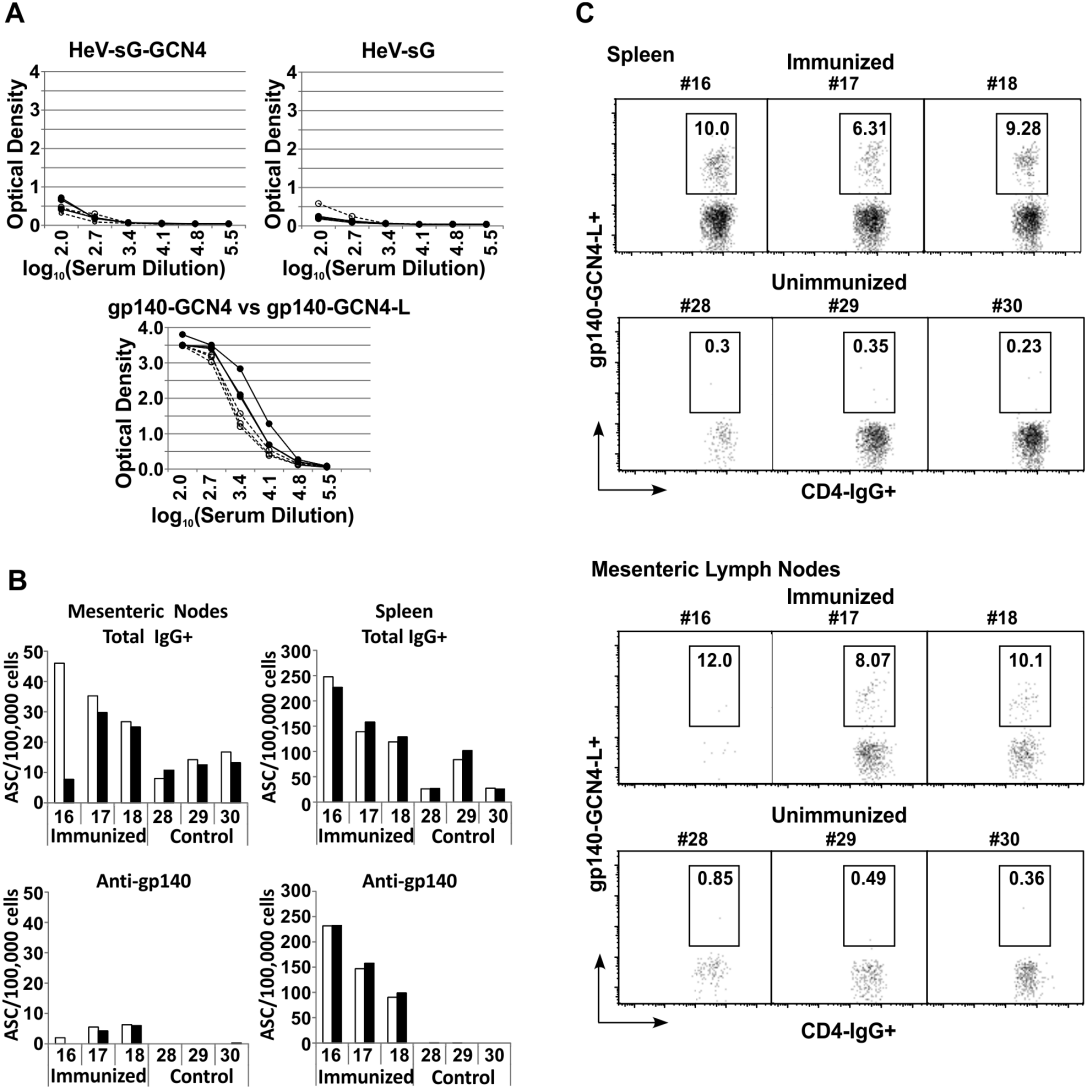
Envelope glycoprotein-specific antibody producing cells in lymph nodes and spleens. (A) Enzyme Linked Immunosorbent Assay (ELISA) of binding of rabbit sera to modified envelope glycoproteins. The presence of antibodies in the three rabbit sera obtained after 6 immunizations against the GCN4 and linker components of gp140-GCN4-L was estimated by testing binding of antibodies in ELISA to proteins with and without GCN4 or linker. Upper panels: sera from immune (•) and control (○) rabbits were tested for antibodies against Hendravirus soluble G protein (HeV-sG) with and without GCN4 carboxy-terminal fusions. No significant difference was noted in binding of immune and control sera to either protein, suggesting lack of antibody response to the GCN4 component. Lower panel: Sera from immune rabbits were tested for binding to R2gp140-GCN4-L (•) and R2gp140-GCN4 (○). No significant difference was noted in binding of the sera to the two proteins (tested by comparing optical densities values at log_10_ (serum dilution) = 3.4, the dilution with maximal splay of results using paired student T test, p = 0.1); results show little or no binding to the linker peptide sequence. (B) Flow cytometry analysis of binding of peripheral blood B cells to R2gp140-GCN4-L. (C) ELISpot assay results on spleens and mesenteric lymph nodes of immune (rabbits 16, 17, and 18) and control (rabbits 28, 29, and 30) rabbits after immunizing dose 7. Cells were suspended by homogenization. Assays were performed in quadruplicate, except for testing of rabbit #16 mesenteric lymph node cells that were tested in single wells. Open bars represent unstimulated cells and black bars represent stimulated cells. No significant differences were noted between the stimulated and unstimulated cell suspensions.

These assays were performed using ELISpot and flow cytometry techniques. In each case we used R2gp140-GCN4-L as antigen for detection of antibody producing cells in tissues of rabbits harvested 10 days after dose 7. To address possible concern that immunizations might have induced significant antibody responses against the GCN4 or linker domains of the fusion protein, we first tested the post 7 dose rabbit sera for binding to proteins with or without these domains in ELISA, as shown in Figure 5A. The two upper panels show relative binding to Hendra virus soluble G protein (HeV-sG) with or without GCN4 fusion domains. In neither case was there greater binding of the HeV-sG by immune than control sera, nor was there greater binding by immune sera of HeV-sGGCN4 than HeV-sG. The lower panel in Figure 5A shows comparative binding of R2gp140-GCN4 and R2gp140-GCN4-L by the immune rabbit sera. There was slightly greater binding of the gp140-GCN4-L protein than the other. However, the difference was not significant, when the results obtained at log_10_ (serum dilution) = 3.4 were compared by paired student T test. This dilution was selected because it displayed maximum splay of results which should have given the highest likelihood of demonstrating a statistically significant difference. Based on these results we conclude that the rabbits had developed little, if any, antibody responses against the GCN4 or linker sequences, and that most of the antibodies were directed against the gp140 sequences.

The results of ELISpot assays of spleen and mesenteric lymph node cells are shown in Figure 5B. Note that the vertical axes of the two panels are on different scales. The numbers of IgG-secreting and anti-gp140-GCN4-L-secreting cells were substantially greater in spleens than lymph nodes. Pre-stimulation of cells with IL-2 (10 ng/ml) + R848 (1 µg/ml) for 72 hours before assay did not result in significant enhancement of numbers of antibody secreting cells detected. The number of total IgG secreting cells detected in the cells from rabbit #16 was greater after stimulation than not, but comparison of all three rabbits by paired T test did not show the difference was significant. There was a significant increase in numbers of antibody secreting cells in both lymph nodes and spleens in the immunized rabbits compared to controls. It appeared that much of the increase in IgG producing cells was accounted for by anti-gp140 secreting cells in the spleen cell suspensions, while a lesser proportion of the increase was due to specific response in the lymph nodes.

Results of assays for spleen and mesenteric lymph node B cells specific for gp140 are shown in Figure 5C. In both cases there was a substantially greater frequency of cells binding gp140-GCN4-L in immunized compared to control rabbits. Overall, these ELISpot and flow cytometry data indicate that vigorous B cell and plasma cell responses were induced in rabbit lymphoid tissues.

## Discussion

This study was conducted to confirm the induction of broadly cross-reactive neutralizing antibodies in the rabbit HIV-1 immunization model, compare relative antigenic reactivity, function, and immunogenicity of different forms of a gp140 Env, develop additional features of the rabbit model, and evaluate the epitope specificity of the neutralizing response. In a previous study we had demonstrated the induction of broadly cross-reactive HIV-1 neutralization in rabbits immunized with the particular strain of HIV-1 gp140 used here, designated R2, in proprietary adjuvant. Limitations of that study were that the R2gp140 used was produced by infection of cells with recombinant vaccinia virus such that the gp140 could only be partially purified from contaminating host cell proteins and anti-human antibody responses developed in response to immunization, and the gp140 that was used was a mixture of higher order forms, mostly dimer. The gp140 itself was constructed to include the gp120 sequence fused to the gp41 ectodomain as a result of elimination of the natural protease cleavage sequences that are used for processing to mature Env in virus. In this study different forms of highly purified gp140 were obtained from stably transformed cell lines for production of the various recombinant proteins. In an effort to mimic the native tertiary structure of Env, trimeric gp140 was constructed by fusion to a GCN4 trimerization domain, and the effect of insertion of a flexible linker sequence between the gp120 and gp41 sequences was also evaluated. The cell line-produced, uncleaved gp140 again was found to be a mixture of dimer and trimer, mainly dimer, while the gp140s with or without the linker but with the GCN4 tail were all trimer. The different forms of gp140 reacted similarly with various mAbs, including direct binding to CD4i mAbs in the absence of CD4, and with CCR5. The comparative immunogenicity with respect to induction of neutralizing antibodies was also similar among the different forms of gp140, although this comparative part of the study might have been more discriminatory if it had been continued for additional immunizations. Broadly cross reactive neutralization was elicited in rabbits that were continued to completion of the immunization protocol. Epitope specificity studies revealed evidence of targeting that did not resemble that of known broadly cross-neutralizing mAbs. Studies of trimeric Env-specific antibody producing cells in spleen and lymph nodes demonstrated robust responses. The results indicate areas of opportunity for future HIV-1 vaccine studies.

The present study was conducted to further evaluate the rabbit model for induction of cross-reactive HIV-1 neutralizing antibodies using R2 Env in AS02_A_ adjuvant that we reported previously. With respect to immunogen design there were two changes planned, producing R2 gp140 using a stably transformed cell line instead of cells acutely infected with recombinant vaccinia virus, and testing different forms of the protein. We were able to produce substantial amounts of the different forms of gp140 using stably transformed 293T cells, and to purify the proteins to high levels. The different forms of gp140 we compared allowed us to evaluate the effect of trimerization and of the presence of a flexible linker sequence between gp120 and gp41 ectodomain sequences on antigenic reactivity. Neither of these types of changes appeared to have major effects on antigenic reactivity as measured by binding to cross-neutralizing human mAbs, binding to CD4i mAbs in the presence or absence of sCD4, or binding to CCR5 in the presence or absence of CD4. Thus, we did not find in vitro evidence that one or another form was likely to be a better immunogen.

De novo induction of potent, broadly cross-reactive HIV-1 neutralizing antibodies in animal models has not been achieved. The use of various adjuvants that ligate TOLL-like receptors as components of Env immunogens has been a common approach to overcome this challenge. In a previous study we employed an adjuvant, AS02_A_, which is an emulsion of monophosphorylated lipid A and QS21, as an adjuvant for soluble R2 Env immunization, and achieved a cross-reactive, low potency response. When planning to confirm and extend that study by conducting the present study, a different adjuvant, designated AS01, was used. This adjuvant is a liposomal formulation including MPL and QS21, and had produced superior results to AS02 in comparative studies of malaria antigen immunization. Thus, the first four immunizations in the present study utilized AS01. After only very weak neutralization responses were obtained, two more immunizations were administered, now using R2 gp140 in AS02, and neutralizing responses were still very weak in all gp140 groups. At that point we elected to discontinue most of the study and give one more immunization to the rabbit group that received the gp140-GCN4-L form of the Env. Surprisingly, this group developed modest potency, cross-reactive neutralizing responses after this last dose. Although we had sought to compare the immunogenicity of the different forms of gp140 tested, we consider it to be unknown whether differential responses would be obtained using an optimal gp140-adjuvant combination.

The neutralizing response that developed in the rabbits that received seven doses of the gp140-GCN4-L immunogen cross-reacted extensively among the strains of HIV-1 tested. The neutralization observed included strains of subtypes A, B, C, and F, as well as strains that were relatively neutralization sensitive (Tier 1 and 1B) and neutralization resistant (Tier 2). There was no neutralization observed against a control virus, VSV. Nor was the neutralizing activity of the sera reduced by extensive absorption of the sera to 293T cells prior to neutralization testing. These data support the interpretation that the neutralizing activity was directed against HIV-1 epitopes and not a non-specific effect on the virus infectivity in the neutralization assay. Evidence to be discussed below showing that markedly different effects of sera were observed in assays using different SF162 strain mutants also demonstrates that the apparent neutralizing activity was not an artifact of the neutralization assay. The results confirm those we obtained previously, that low to modest potency cross-reactive HIV-1 neutralizing responses can be obtained in the rabbit model.

The epitope specificity of the neutralizing responses observed was probed to look for similarity to know broadly cross-reactive neutralizing human mAbs. The neutralizing activity in the rabbit sera was unlike that of the human mAbs VRC01 and VRC03 that target the CD4 binding site on HIV-1in that it was not blocked by the soluble protein RSC3 that inhibits the neutralizing activity of these mAbs. Neither did we find evidence indicating that the rabbits developed anti-coreceptor binding site antibodies: there was no enhancement of neutralization of HIV-2 by the immune rabbit sera in the presence of sCD4. There are two major groups of broadly cross-neutralizing mAbs that recognize different conformational, glycan-dependent epitopes. These groups are represented in our study by the mAbs PGT121 and PGT126, on the one hand, and PG9 and PG16, on the other, and are distinguished by the specific glycans upon which they depend for epitope recognition. Not only was the presence of either of these glycans not required for neutralization by our immune rabbit sera, the neutralizing effects of the sera were dramatically increased when both of these glycans were absent. Antibodies against the membrane proximal external region of gp41 may exhibit broadly cross-reactive neutralization. We did not test the rabbit sera for antibodies with that specificity, but consider it very unlikely that such antibodies were induced by the gp140 used here, since they have not been induced by similar antigens in other studies. The rabbits did develop vigorous responses against the V1/V2 region represented in the gp70-V1V2 peptide we tested in ELISA. Antibodies against variable regions of the Env could contribute to the neutralizing activity in our rabbit sera, but direct evidence of such was not obtained in this study.

A major and enduring challenge in HIV vaccine development is induction of neutralizing antibody responses that are both potent and durable. To date, neutralizing responses that have been induced in animal models have been of limited potency and non-durable, raising questions about the extent to which B cells that produce immunoglobulin molecules that neutralize are actually induced by the immunogens. The antibodies we induced in this study did exhibit cross-reactive neutralization, but the potency of the responses was limited. To evaluate whether the B cell responses of the rabbits were weakly induced we studied frequencies of B cells or plasma cells in spleens producing antibodies that bound soluble R2 gp140-GCN4-L trimer. To the extent that this molecule resembles the authentic, functional Env spike, antibodies that bind to the trimer are likely to be neutralizing. ELISpot assays were conducted to measure numbers of antibody secreting plasma cells in spleens. Both total Ig secreting cells and R2 gp140 trimer binding Ig secreting cells were markedly increased by immunization, and the majority of Ig secreting cells appeared to be specific for the gp140 trimer. By flow cytometry analyses, total numbers of B cells displaying surface Ig that bound to gp140-GCN4-L trimer were also substantially increased. The results demonstrate that preimmune B cells were effectively induced to produce immunogen-specific Ig and to differentiate into plasma cells. The results do not clarify the reasons for the lack of potency or durability of the neutralizing responses. Possibly, some of the trimer-binding antibodies were non-neutralizing or the initial vigorous responses observed were not sustained and did not evolve into mature B memory and long term plasma cell responses. Additional studies will be needed to resolve explain the problems with durability of the responses.

Further, the availability of a small animal model for induction of neutralizing antibody responses against HIV-1 would be a useful advancement for the field of HIV vaccine development. Here we demonstrate the biophysical and antigenic characteristics of several different forms of highly purified R2 gp140, and demonstrate the ability of gp140-GCN4-L, gp140 trimer with a flexible linker between the gp120 and gp41 ectodomain sequences, to induce a broadly cross-reactive neutralizing response in outbred NZW rabbits. The breadth of the neutralizing cross-reactivity was similar to that reported previously in study of a less purified form of R2 gp140. Studies of the specificity of the neutralization did not indicate that the epitope(s) targeted corresponded to well-known humAbs with broad neutralizing cross-reactivity; better definition of the specificity of the response may depend upon development of neutralizing mAbs from rabbits with such responses. Efforts to develop responses of greater potency and durability in this model are warranted.

### 

#### Disclaimer

The views expressed are those of the authors and do not necessarily reflect the official views of the Uniformed Services University of the Health Sciences or the Department of Defense.

## References

[1] Rerks-Ngarm S, Pitisuttithum P, Nitayaphan S, Kaewkungwal J, Chiu J, et al (2009) Vaccination with ALVAC and AIDSVAX to prevent HIV-1 infection in Thailand. N Engl J Med 361: 2209–2220. 19843557 10.1056/NEJMoa0908492

[2] Kim JH, Rerks-Ngarm S, Excler JL, Michael NL (2010) HIV vaccines: lessons learned and the way forward. Curr Opin HIV AIDS 5: 428–434. 20978385 10.1097/COH.0b013e32833d17ac PMC2990218

[3] Buchbinder SP, Mehrotra DV, Duerr A, Fitzgerald DW, Mogg R, et al (2008) Efficacy assessment of a cell-mediated immunity HIV-1 vaccine (the Step Study): a double-blind, randomised, placebo-controlled, test-of-concept trial. Lancet 372: 1881–1893. 19012954 10.1016/S0140-6736(08)61591-3 PMC2721012

[4] Walker LM, Simek MD, Priddy F, Gach JS, Wagner D, et al (2010) A limited number of antibody specificities mediate broad and potent serum neutralization in selected HIV-1 infected individuals. PLoS Pathog 6: e1001028. 20700449 10.1371/journal.ppat.1001028 PMC2916884

[5] Walker LM, Huber M, Doores KJ, Falkowska E, Pejchal R, et al (2011) Broad neutralization coverage of HIV by multiple highly potent antibodies. Nature 477: 466–470. 21849977 10.1038/nature10373 PMC3393110

[6] Huang J, Ofek G, Laub L, Louder MK, Doria-Rose NA, et al (2012) Broad and potent neutralization of HIV-1 by a gp41-specific human antibody. Nature 491: 406–412. 23151583 10.1038/nature11544 PMC4854285

[7] Wu X, Yang ZY, Li Y, Hogerkorp CM (2010) Schief WR, et al (2010) Rational design of envelope identifies broadly neutralizing human monoclonal antibodies to HIV-1. Science 329: 856–861. 20616233 10.1126/science.1187659 PMC2965066

[8] Scheid JF, Mouquet H, Feldhahn N, Seaman MS, Velinzon K, et al (2009) Broad diversity of neutralizing antibodies isolated from memory B cells in HIV-infected individuals. Nature 458: 636–640. 19287373 10.1038/nature07930

[9] Kwong PD, Mascola JR, Nabel GJ (2012) The changing face of HIV vaccine research. J Int AIDS Soc 15: 17407. 22789610 10.7448/IAS.15.2.17407 PMC3499796

[10] Dong M, Zhang PF, Grieder F, Lee J, Krishnamurthy G, et al (2003) Induction of primary virus-cross-reactive human immunodeficiency virus type 1-neutralizing antibodies in small animals by using an alphavirus-derived in vivo expression system. J Virol 77: 3119–3130. 12584337 10.1128/JVI.77.5.3119-3130.2003 PMC149731

[11] Quinnan GV Jr, Yu XF, Lewis MG, Zhang PF, Sutter G, et al (2005) Protection of rhesus monkeys against infection with minimally pathogenic simian-human immunodeficiency virus: correlations with neutralizing antibodies and cytotoxic T cells. J Virol 79: 3358–3369. 15731230 10.1128/JVI.79.6.3358-3369.2005 PMC1075715

[12] Zhang MY, Borges AR, Ptak RG, Wang Y, Dimitrov AS, et al (2010) Potent and broad neutralizing activity of a single chain antibody fragment against cell-free and cell-associated HIV-1. MAbs 2: 266–274. 20305395 10.4161/mabs.2.3.11416 PMC2881253

[13] Zhang PF, Bouma P, Park EJ, Margolick JB, Robinson JE, et al (2002) A variable region 3 (V3) mutation determines a global neutralization phenotype and CD4-independent infectivity of a human immunodeficiency virus type 1 envelope associated with a broadly cross-reactive, primary virus-neutralizing antibody response. J Virol 76: 644–655. 11752155 10.1128/JVI.76.2.644-655.2002 PMC136808

[14] Zhang PF, Cham F, Dong M, Choudhary A, Bouma P, et al (2007) Extensively cross-reactive anti-HIV-1 neutralizing antibodies induced by gp140 immunization. Proc Natl Acad Sci U S A 104: 10193–10198. 17540729 10.1073/pnas.0608635104 PMC1885220

[15] Selvarajah S, Puffer BA, Lee FH, Zhu P, Li Y, et al (2008) Focused dampening of antibody response to the immunodominant variable loops by engineered soluble gp140. AIDS Res Hum Retroviruses 24: 301–314. 18284327 10.1089/aid.2007.0158

[16] Yang X, Florin L, Farzan M, Kolchinsky P, Kwong PD, et al (2000) Modifications that stabilize human immunodeficiency virus envelope glycoprotein trimers in solution. J Virol 74: 4746–4754. 10775613 10.1128/jvi.74.10.4746-4754.2000 PMC111997

[17] Chow YH, Wei OL, Phogat S, Sidorov IA, Fouts TR, et al (2002) Conserved structures exposed in HIV-1 envelope glycoproteins stabilized by flexible linkers as potent entry inhibitors and potential immunogens. Biochemistry 41: 7176–7182. 12033952 10.1021/bi025646d

[18] Chan YP, Lu M, Dutta S, Yan L, Barr J, et al (2012) Biochemical, conformational, and immunogenic analysis of soluble trimeric forms of henipavirus fusion glycoproteins. J Virol 86: 11457–11471. 22915804 10.1128/JVI.01318-12 PMC3486283

[19] Pallister J, Middleton D, Wang LF, Klein R, Haining J, et al (2011) A recombinant Hendra virus G glycoprotein-based subunit vaccine protects ferrets from lethal Hendra virus challenge. Vaccine 29: 5623–5630. 21689706 10.1016/j.vaccine.2011.06.015 PMC3153950

[20] Xiang SH, Doka N, Choudhary RK, Sodroski J, Robinson JE (2002) Characterization of CD4-induced epitopes on the HIV type 1 gp120 envelope glycoprotein recognized by neutralizing human monoclonal antibodies. AIDS Res Hum Retroviruses 18: 1207–1217. 12487827 10.1089/08892220260387959

[21] Thali M, Moore JP, Furman C, Charles M, Ho DD, et al (1993) Characterization of conserved human immunodeficiency virus type 1 gp120 neutralization epitopes exposed upon gp120-CD4 binding. J Virol 67: 3978–3988. 7685405 10.1128/jvi.67.7.3978-3988.1993 PMC237765

[22] Huang CC, Venturi M, Majeed S, Moore MJ, Phogat S, et al (2004) Structural basis of tyrosine sulfation and VH-gene usage in antibodies that recognize the HIV type 1 coreceptor-binding site on gp120. Proc Natl Acad Sci U S A 101: 2706–2711. 14981267 10.1073/pnas.0308527100 PMC365685

[23] Wyatt R, Moore J, Accola M, Desjardin E, Robinson J, et al (1995) Involvement of the V1/V2 variable loop structure in the exposure of human immunodeficiency virus type 1 gp120 epitopes induced by receptor binding. J Virol 69: 5723–5733. 7543586 10.1128/jvi.69.9.5723-5733.1995 PMC189432

[24] Gershoni JM, Denisova G, Raviv D, Smorodinsky NI, Buyaner D (1993) HIV binding to its receptor creates specific epitopes for the CD4/gp120 complex. Faseb J 7: 1185–1187. 7690724 10.1096/fasebj.7.12.7690724

[25] Lee S, Peden K, Dimitrov DS, Broder CC, Manischewitz J, et al (1997) Enhancement of human immunodeficiency virus type 1 envelope-mediated fusion by a CD4-gp120 complex-specific monoclonal antibody. J Virol 71: 6037–6043. 9223495 10.1128/jvi.71.8.6037-6043.1997 PMC191861

[26] Zhang MY, Xiao X, Sidorov IA, Choudhry V, Cham F, et al (2004) Identification and characterization of a new cross-reactive human immunodeficiency virus type 1-neutralizing human monoclonal antibody. J Virol 78: 9233–9242. 15308718 10.1128/JVI.78.17.9233-9242.2004 PMC506938

[27] Mirzabekov T, Bannert N, Farzan M, Hofmann W, Kolchinsky P, et al (1999) Enhanced expression, native purification, and characterization of CCR5, a principal HIV-1 coreceptor. J Biol Chem 274: 28745–28750. 10497246 10.1074/jbc.274.40.28745

[28] Ohagen A, Li L, Rosenzweig A, Gabuzda D (2000) Cell-dependent mechanisms restrict the HIV type 1 coreceptor activity of US28, a chemokine receptor homolog encoded by human cytomegalovirus. AIDS Res Hum Retroviruses 16: 27–35. 10628814 10.1089/088922200309575

[29] Davenport TM, Friend D, Ellingson K, Xu H, Caldwell Z, et al (2011) Binding interactions between soluble HIV envelope glycoproteins and quaternary-structure-specific monoclonal antibodies PG9 and PG16. J Virol 85: 7095–7107. 21543501 10.1128/JVI.00411-11 PMC3126577

[30] Li M, Gao F, Mascola JR, Stamatatos L, Polonis VR, et al (2005) Human immunodeficiency virus type 1 env clones from acute and early subtype B infections for standardized assessments of vaccine-elicited neutralizing antibodies. J Virol 79: 10108–10125. 16051804 10.1128/JVI.79.16.10108-10125.2005 PMC1182643

[31] Li M, Salazar-Gonzalez JF, Derdeyn CA, Morris L, Williamson C, et al (2006) Genetic and neutralization properties of subtype C human immunodeficiency virus type 1 molecular env clones from acute and early heterosexually acquired infections in Southern Africa. J Virol 80: 11776–11790. 16971434 10.1128/JVI.01730-06 PMC1642599

[32] Seaman MS, Janes H, Hawkins N, Grandpre LE, Devoy C, et al (2010) Tiered categorization of a diverse panel of HIV-1 Env pseudoviruses for assessment of neutralizing antibodies. J Virol 84: 1439–1452. 19939925 10.1128/JVI.02108-09 PMC2812321

[33] Lynch RM, Tran L, Louder MK, Schmidt SD, Cohen M, et al (2012) The development of CD4 binding site antibodies during HIV-1 infection. J Virol 86: 7588–7595. 22573869 10.1128/JVI.00734-12 PMC3416294

[34] Zhang MY, Wang Y, Mankowski MK, Ptak RG, Dimitrov DS (2009) Cross-reactive HIV-1-neutralizing activity of serum IgG from a rabbit immunized with gp41 fused to IgG1 Fc: possible role of the prolonged half-life of the immunogen. Vaccine 27: 857–863. 19084043 10.1016/j.vaccine.2008.11.083 PMC3399430

[35] Decker JM, Bibollet-Ruche F, Wei X, Wang S, Levy DN, et al (2005) Antigenic conservation and immunogenicity of the HIV coreceptor binding site. J Exp Med 201: 1407–1419. 15867093 10.1084/jem.20042510 PMC2213183

[36] Haynes BF, Gilbert PB, McElrath MJ, Zolla-Pazner S, Tomaras GD, et al (2012) Immune-correlates analysis of an HIV-1 vaccine efficacy trial. N Engl J Med 366: 1275–1286. 22475592 10.1056/NEJMoa1113425 PMC3371689

[37] Pinter A, Honnen WJ, He Y, Gorny MK, Zolla-Pazner S, et al (2004) The V1/V2 domain of gp120 is a global regulator of the sensitivity of primary human immunodeficiency virus type 1 isolates to neutralization by antibodies commonly induced upon infection. J Virol 78: 5205–5215. 15113902 10.1128/JVI.78.10.5205-5215.2004 PMC400352

